# The findings of corneal specular microscopy in patients with type-2 diabetes mellitus

**DOI:** 10.1186/s12886-020-01488-9

**Published:** 2020-06-03

**Authors:** Nurdan Gamze Taşlı, Erel Icel, Yücel Karakurt, Turgay Ucak, Adem Ugurlu, Hayati Yilmaz, Emin Murat Akbas

**Affiliations:** Erzincan Binali Yildirim University Faculty of Medicine Department of Ophthalmology, 24100 Erzincan, Turkey

**Keywords:** Specular microscopy, Corneal endothelial cells, Diabetes mellitus, Microalbuminuria

## Abstract

**Background:**

We aimed to compare the morphological characteristics of corneal endothelial cells in type 2 diabetic patients and age-matched healthy subjects by specular microscopy.

We also aimed to determine the association of corneal morphological features with the general characteristics and laboratory data of diabetic patients, including disease duration, haemoglobin A1c (HbA1c) levels and urine albumin creatinine ratio.

**Methods:**

A total of 195 diabetic patients and 100 healthy controls were enrolled in the study. All participants underwent a complete ophthalmological examination. Corneal endothelial measurements were performed using a noncontact specular microscopy. Laboratory data including serum fasting glucose, haemoglobin A1c levels, creatinine levels, and the urinary albumin-to-creatinine ratio were recorded. Diabetic patients were further subdivided into 3 groups according to the presence and stage of diabetic retinopathy. Specular microscopy findings and central corneal thickness of all patients were compared.

**Results:**

The ECD and hexagonal cell ratio were significantly lower, while the average cell size, CV%, and central corneal thickness were determined to be significantly higher in diabetic patients than in healthy controls (*p* = 0.001). With the presence and advancement of diabetic retinopathy, the ECD and hexagonal cell ratio decreased, while the average cell size, CV%, and central corneal thickness increased. When correlation analysis was performed between corneal morphological features and laboratory data of diabetic patients, ECD showed a significant negative correlation with diabetes duration (*p* = 0.028). HbA1c levels, urinary albumin-creatinine ratio (*p* = 0.041), average cell size and CV showed a positive correlation with these parameters.

**Conclusion:**

In conclusion, keratopathy is an important complication of type 2 diabetes. With an increase in the stage of diabetic retinopathy, alterations in corneal findings also increased. In that respect, we can suggest that keratopathy should be evaluated more cautiously in diabetic patients.

## Background

Diabetes mellitus (DM) is associated with damage and dysfunction of various organs, especially the eyes, kidneys, nerves, heart, and blood vessels [1]. Diabetic retinopathy (DR) is one of the most important causes of blindness and affects approximately 40% of all diabetic patients [2]. In general, diabetes affects both vascular and neural cells of the retina and is characterized by alterations of the retinal microvasculature [3, 4].

Diabetes also affects the cornea, and diabetic keratopathy is another ocular complication of diabetes that affects approximately half of diabetes patients [5–7]. However, the data regarding diabetic keratopathy are limited and conflicting in the literature [8, 9]. In diabetic patients, cellular dysfunction in the cornea may cause defects in repair mechanisms.

Diabetic nephropathy is an additional important complication of type 2 DM and may be fatal. The main stages of diabetic nephropathy are hyperfiltration, microalbuminuria, and frank proteinuria [10]. Microalbuminuria is not only an early marker of diabetic nephropathy but is also considered an indicator of morbidity and mortality in diabetic patients [11, 12].

Our study aimed to compare the morphological characteristics of corneal endothelial cells in type 2 diabetic patients and age-matched healthy subjects by specular microscopy. We also aimed to analyse alterations in the cornea in diabetic patients with advanced-stage diabetic retinopathy. Moreover, we aimed to determine the association of corneal morphological features with the general characteristics and laboratory data of diabetic patients, including disease duration, haemoglobin A1c (HbA1c) levels and urine albumin creatinine ratio. In this way, we aimed to analyse the association of the degree of alterations in the diabetic cornea with the stage of diabetic nephropathy, which is a prognostic marker in diabetes patients.

## Methods

This prospective study was performed at Erzincan Binali Yıldırım University between 12/2017 and 06/2018. The study was approved by the local ethics committee, and informed consent was obtained from all participants. In total, 195 diabetic patients and 100 healthy, control participants were concomitantly enrolled in the study.

The exclusion criteria were the use of contact lenses; presence of dry eye disease; history of any ocular diseases including corneal scar, cataract or glaucoma; and any history of previous ocular surgery. All participants underwent a complete ophthalmic evaluation, including slit-lamp biomicroscopic examination, Goldman applanation tonometry, and fundoscopy with pupil dilation. Grading of DR was based on the International Clinical Diabetic Retinopathy Disease Severity Scale as without DR, with background DR or with proliferative DR [13].

The patients who were diagnosed with type 2 diabetes and under follow-up in our hospital were included in the study. The diagnosis of diabetes was based on the ADA (American Diabetes Association) criteria; fasting blood glucose ≥126 mg/dl on two separate occasions, random blood glucose (RBG) ≥200 mg/dl with symptoms or 2-h plasma glucose ≥200 mg/dl [14].

Diabetic patients were further subdivided into 3 groups based on the presence and stage of diabetic retinopathy as patients without DR, with background DR, and with proliferative DR After 8 h of fasting, serum samples of study participants were obtained to analyse serum glucose, HbA1c, blood urea nitrogen and creatinine levels.

The levels of albumin and creatinine were studied from urine specimens using an immunoturbidimetric method. A urine albumin/creatinine ratio between 0 and 30 mg/g was accepted as normoalbuminuria, and 30 mg/g was accepted as microalbuminuria [15].

One eye of all participants was examined. Endothelial cell density (ECD), average cell size, percentage of hexagonal cells, coefficient of variation (CV) in cell size, and central corneal thickness (CCT) of all patients were measured using a noncontact specular microscopy (CEM-530 Specular Microscope, NIDEK, Japan) device.

All measurements were performed in the morning between 9:00 and 11.00 am by the same skilled ophthalmologist.

### Statistical analysis

Data analysis was performed using SPSS software (version 21.0, SPSS, Inc.). The normality of the distribution of the data was analysed with the Shapiro–Wilk test, and since all data were normally distributed, parametric tests were used. For the comparison of two groups, independent samples t-test and chi-square test were performed. Pearson correlation analysis was used to examine the correlation of corneal findings with the clinical characteristics of diabetic patients, including disease duration, HbA1c levels and microalbuminuria. *p* values less than 0.05 were considered significant.

## Results

In total, 195 eyes of 195 patients (87 male, 108 female) with type 2 diabetes mellitus were included in the study. In the control group, 100 eyes of 100 (37 male, 63 female) healthy cases with normal fasting blood glucose and HbA1c levels were included. There was no significant difference between groups regarding sex (*p* = 0.07) or age (*p* = 0.141) (Table 1).

**Table 1:** Demographic features of study participants

	Diabetic group (n: 195)	Healthy controls (n: 100)	p
**Gender (M/F)**	87/108	37/63	0.079
**Age (years)**	61.38 ± 8.449	59.60 ± 7.975	0.141

*M* male, *F* female

Fasting blood glucose levels, HbA1c and urinary albumin-creatinine ratio levels were significantly higher in the diabetic group (*p* = 0.001). The ECD and hexagonal cell ratio were significantly lower, while the average cell size, CV%, and CCT were determined to be significantly higher in diabetic patients than in healthy controls (Table 2).

**Table 2:** Comparison of laboratory data and ocular findings of study groups

	Diabetic group (n: 195)	Healthy controls (n: 100)	p
**Fasting Blood Glucose (mg/dl)**	176.06 ± 55.52	94.37 ± 11.08	**0.001**
**Hemoglobin A 1c (%)**	8.14 ± 1.60	5.26 ± 0.72	**0.001**
**Blood urea nitrogen**	37.77 ± 12.94	30.11 ± 6.88	**0.001**
**Creatinine**	0.98 ± 0.32	0.81 ± 0.27	**0.001**
**Urine albumin-creatinine ratio (mg/g·Cre)**	54.3473 ± 59.82	2.60 ± 2.08	**0.001**
**Endothelial Cell Density (cells/m**^**2**^**)**	2427.94 ± 427.10	2615.56 ± 106.80	**0.001**
**Average cell size (μm**^**2**^**)**	419.69 ± 71.98	374.74 ± 14.11	**0.001**
**Coefficient of Variation (%)**	32.37 ± 6.16	30.29 ± 4.08	**0.001**
**Hexagonal cell ratio (%)**	66.94 ± 7.17	78.48 ± 6.56	**0.001**
**Central corneal thickness (μ)**	539.01 ± 41.19	525.90 ± 28.79	**0.001**

**Fig. 1:**
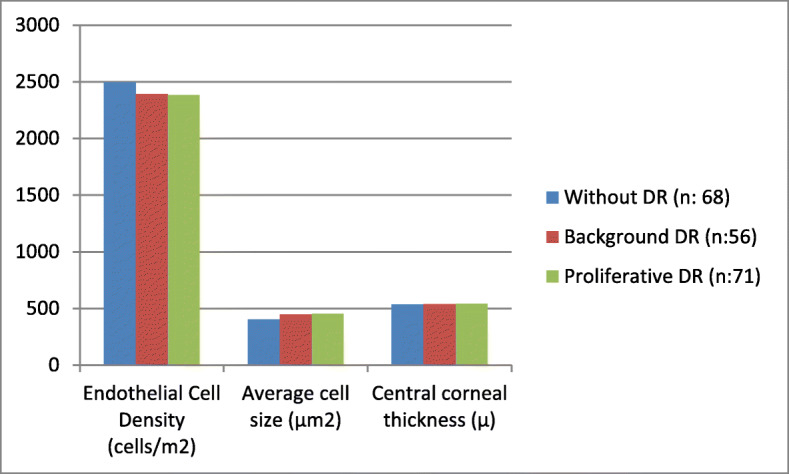
Comparison of ocular findings of three subgroups of diabetic patients

Demographic features, ocular findings and laboratory data of diabetic patients are summarized in Table 3. There was no significant difference between groups regarding sex or age. With the presence and advancement of DR, the ECD and hexagonal cell ratio decreased, while the average cell size, CV%, and CCT increased (Table 3) (Fig. 1).

**Table 3:** Demographic features, laboratory data and ocular findings of three subgroups of diabetic patients

	Without DR (n: 68)	Background DR (n:56)	Proliferative DR (n:71)	P1	P2	P3
**Gender (M/F)**	28/40	24/32	35/36	0.56	0.14	0.24
**Age (years)**	61.50 ± 7.791	63.77 ± 8.17	62.37 ± 7.07	0.145	0.124	0.247
**Diabetes duration (years)**	5.32 ± 2.06	9.07 ± 3.92	13.25 ± 4.33	**0.001**	**0.001**	**0.001**
**Fasting Blood Glucose (mg/dl)**	139.33 ± 36.50	189.53 ± 50.06	203.51 ± 57.91	**0.001**	**0.001**	**0.004**
**Hemoglobin A 1c (%)**	7.02 ± 1.15	8.46 ± 1.41	9.13 ± 1.45	**0.001**	**0.001**	**0.001**
**Blood urea nitrogen**	30.56 ± 9.11	39.81 ± 12.02	43.92 ± 13.97	**0.001**	**0.001**	**0.001**
**Creatinine**	0.80 ± 0.27	1.01 ± 0.31	1.17 ± 0.26	**0.001**	**0.001**	**0.001**
**Urine albumin-creatinine ratio (mg/g·Cre)**	9.87 ± 4.86	52.47 ± 42.72	113.30 ± 64.58	**0.001**	**0.001**	**0.001**
**Endothelial Cell Density (cells/m**^**2**^**)**	2495.83 ± 365.39	2392.28 ± 462.73	2384.06 ± 432.98	**0.043**	**0.021**	0.872
**Average cell size (μm**^**2**^**)**	403.22 ± 64.22	446.92 ± 79.08	452.16 ± 62.11	**0.012**	**0.009**	0.308
**Coefficient of Variation (%)**	30.04 ± 5.29	31.34 ± 5.46	33.58 ± 5.58	**0.044**	**0.004**	0.408
**Hexagonal cell ratio (%)**	66.16 ± 6.43	65.20 ± 5.80	64.60 ± 7.84	0.437	0.412	0.116
**Central corneal thickness (μ)**	536.29 ± 51.68	538.84 ± 34.52	541.99 ± 33.21	0.467	0.278	0.654

*M* male, *F* female; *p1* p value of the comparison between Without DR and Background DR groups; *p2* p value of the comparison between Without DR and Proliferative DR groups; *p3 p* value of the comparison between Background DR and Proliferative DR groups

**Fig. 2:**
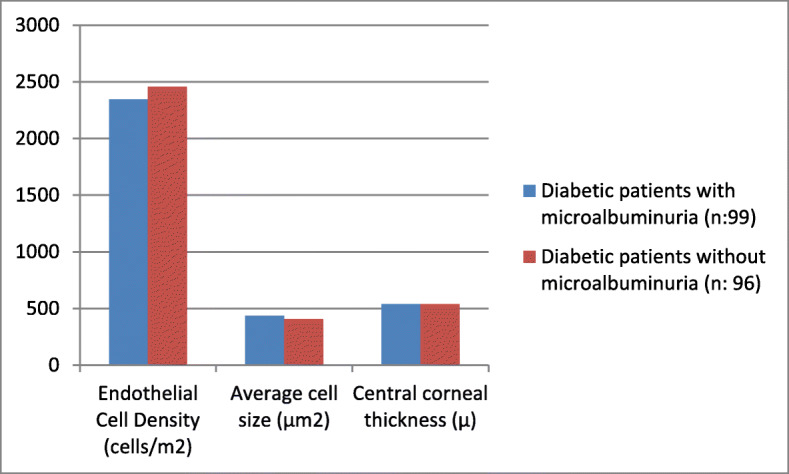
Comparison of ocular findings between two groups

Diabetic patients with (n:99) or without (n:96) microalbuminuria were compared in regard to demographic features and corneal findings and the data are summarized in Table 4 (Fig. 2). There was no significant difference between groups regarding sex or age. In diabetic patients with microalbuminuria, ECD was significantly lower (2344.25 ± 159.75 vs. 2458.44 ± 183.92 cells/m^2^; *p* = 0.004**)**, while average cell size (436.64 ± 38.50 vs. 405.86 ± 77.73; *p* = 0.038) and CV% (33.28 ± 5.82 vs. 31.45 ± 6.41; p = 0.004) were significantly higher.

**Table 4:** Comparison of corneal findings in diabetic patients with or without microalbuminuria

	Diabetic patients with microalbuminuria (n:99)	Diabetic patients without microalbuminuria (n: 96)	p
**Gender (M/F)**	42/57	45/51	0.25
**Age (years)**	61.74 ± 8.86	63.01 ± 7.16	0.11
**Diabetes duration (years)**	11.47 ± 4.37	6.51 ± 3.67	**0.001**
**Endothelial Cell Density (cells/m**^**2**^**)**	2344.25 ± 159.75	2458.44 ± 183.92	**0.004**
**Average cell size (μm**^**2**^**)**	436.64 ± 38.50	405.86 ± 77.73	**0.038**
**Coefficient of Variation (%)**	33.28 ± 5.82	31.45 ± 6.41	**0.004**
**Hexagonal cell ratio (%)**	66.58 ± 5.82	67.36 ± 8.37	0.293
**Central corneal thickness (μ)**	539.29 ± 33.04	538.73 ± 47.96	0.891

When correlation analysis was performed between the corneal morphological features and general characteristics and laboratory data of diabetic patients, ECD showed a significant negative correlation with diabetes duration (*p* = 0.028), HbA1c levels (*p* = 0.033) and urinary albumin-creatinine ratio (*p* = 0.041), and average cell size and CV showed a positive correlation with these parameters (Table 5).

**Table 5:** Results of correlation analysis performed between corneal morphological features and general characteristics and laboratory data of diabetic patients

	Diabetes duration		Hgb A1c		Creatinine		Urine albumin-creatinine ratio	
	r	p	r	p	r	p	r	p
**Endothelial Cell Density (cells/m**^**2**^**)**	−0.111	**0.028**	−0.211	**0.033**	−0.063	0.21	−0.122	**0.041**
**Average cell size (μm**^**2**^**)**	0.141	**0.005**	0.331	**0.011**	0.088	0.083	0.111	**0.029**
**Coefficient of Variation (%)**	0.094	**0.044**	0.097	**0.043**	0.042	0.461	0.121	**0.017**
**Hexagonal cell ratio (%)**	−0.058	0.093	−0.056	0.072	−0.012	0.552	−0.034	0.198

## Discussion

In this study, we identified significantly decreased ECD and percentage of hexagonal cells, elevated average cell size and coefficient of variation in diabetic patients compared with the same parameters in healthy controls. Moreover, with an increase in the stage of DR, ECD and the percentage of hexagonal cells decreased while central corneal thickness increased. In this study, we determined a significant negative correlation between ECD and diabetes duration, HbA1c levels and the urinary albumin-creatinine ratio of patients in the diabetic group. To the best of our knowledge, this is the first study in the literature evaluating the association of corneal morphological parameters with different microvascular complications (retinopathy and nephropathy) of diabetes. In this study, unlike other studies, retinopathy nephropathy and keratopathy were evaluated together. The relationship between the severity of retinopathy and the cornea was evaluated, and a significant relationship was found.

The associations of corneal complications and instability of corneal endothelium in diabetic patients with excessive sorbitol accumulation in the corneal endothelium and accumulation of advanced glycation end products in the epithelial basement membrane have been known for years [16, 17]. However, the data in the literature evaluating corneal morphological parameters in diabetic patients are conflicting, and these parameters may be associated with ethnic differences. In this study, we determined a significant decrease in ECD values in diabetic patients compared with the values in age-matched controls, and there was a negative correlation between ECD and diabetes duration. The cornea is incapable of mitosis, and corneal endothelial cells have no regenerative capacity. For that reason, we can suggest that with an elongation in diabetic duration, the decrease in ECD cannot be restored. The only compensation mechanism at that point is the increased cellular pleomorphism and a decrease in the percentage of hexagonal cells. We also identified a correlation between average cell size and CV with diabetes duration, but there was no significant correlation between hexagonal cell percentage and diabetes duration or HbA1c levels in our study. Similar to our results, Lee et al. [18] also reported decreased ECD and hexagonality in diabetic eyes with thicker corneas compared with the same parameters in healthy controls. Moreover, they also determined an augmentation in corneal morphological abnormalities in patients with a diabetic duration of over 10 years. El-Agamy et al. [19] reported that ECD was significantly lower while CV was significantly higher in diabetic patients, but the differences in hexagonal cell percentage and CCT were not significantly different between diabetic patients and healthy controls. In a population-based cross-sectional study, Sudhir et al. [20] reported that ECD was significantly lower in diabetic patients than in controls, but there was no significant difference between groups regarding hexagonality % or CV of cell size.

Islam QU et al. [21] reported that the mean ECD was significantly lower in diabetic patients than in age-matched healthy controls, but the mean average cell size, CV and hexagonality were not significantly different between the two groups. Similar to our results, the duration of diabetes was significantly correlated with ECD and hexagonality. Choo et al. [22] reported that ECD and hexagonality significantly decreased, while average cell size and CV increased significantly in diabetic patients. However, they determined that there was no significant alteration in the CCT of patients with diabetes and that there was no correlation between corneal endothelial findings and the duration of diabetes, HbA1c level or severity of diabetic retinopathy. Leem et al. [23] reported that central corneal thickness was increased and ECD was decreased in patients with diabetes mellitus, and contact lens usage also significantly affected corneal morphology in diabetic patients. In our study, we did not include patients using contact lenses.

In a prospective study, Storr-Paulsen et al. [24] reported that CCT was significantly higher in diabetic patients, but there were no significant differences between type 2 diabetic patients with good glycaemic control and nondiabetic control subjects in ECD, hexagonality or CV values. However, they also reported a decrease in ECD values with an increase in HbA1c levels. In our study, the mean HbA1c of the diabetic group was not low (8.14 ± 1.60), and we also reported a negative correlation between HbA1c and ECD.

Microalbuminuria is an indicator of early cardiovascular death and progressive renal disease in diabetic patients [25, 26]. In this study, we determined a significant correlation between the urine albumin-creatinine ratio and ECD, average cell size and CV. To the best of our knowledge, this is the first study in the literature evaluating the association of microalbuminuria with ocular findings. In that respect, we can suggest that keratopathy should be evaluated in diabetic patients with more awareness.

Age is defined as the most important factor in evaluating corneal morphology, and it is known that with age, ECD decreases and the corneal endothelium compensate by increasing the size. In this study, the diabetic and control groups were age-matched. However, in subgroups of diabetes, age could not be matched, which may have affected the results and is one of the limitations of this study. There are also some other limitations of this study that should be mentioned. Although the determination of microalbuminuria with a single random urine sample is highly sensitive and specific, the gold standard in the diagnosis of microalbuminuria is an analysis of 24-h urine collection.

The corneal endothelium can be evaluated by specular microscopy. In the biomicroscopic examination of the eye, the fact that the cornea is transparent does not mean that the endothelium is normal. It may be useful for early diagnosis to direct patients with endothelial problems to nephrologists for clinical evaluation of nephropathy. In line with the data we obtained at the end of this study, diabetes mellitus reduces corneal endothelial functional reserve. Reduced endothelial reserve increases the risk of corneal endothelial damage in intraocular surgery. Additionally, the risk of corneal decompensation should be kept in mind in diabetic patients as the severity of the disease increases. In diabetic patients with diabetic retinopathy and with nephropathy, caution should be exercised in terms of endothelial decompensation. To minimize endothelial damage during cataract surgery and other surgeries, if necessary, endothelial protective manoeuvres should be performed, and preoperative specular microscopy findings should be carefully examined in these patients.

## Conclusions

In conclusion, keratopathy with decreased ECD and percentage of hexagonal cells and increased coefficient of variation is an important complication of type 2 diabetes. With an increase in the stage of DR, alterations in corneal findings also increased. There was a significant correlation between the urine albumin-creatinine ratio and ECD, average cell size and CV in diabetic patients. In that respect, we can suggest that keratopathy should be evaluated more cautiously in diabetic patients. Larger, prospective studies are warranted to define the role of keratopathy in the long-term outcomes of diabetic patients.

## Data Availability

All generated or analysed data in this study are included in the supporting file. The datasets of the current study are available from the corresponding author on reasonable request.

## Abbreviations

DM: Diabetes Mellitus
DR: Diabetic retinopathy
HbA1c: Haemoglobin A1c
ADA: American Diabetes Association
RBG: Random blood glucose
ECD: Endothelial cell density
CV: Coefficient of variation
CCT: Central corneal thickness
